# *EGFR* 20外显子插入突变非小细胞肺癌规范化诊疗中国专家共识（2024版）

**DOI:** 10.3779/j.issn.1009-3419.2024.102.27

**Published:** 2024-07-20

**Authors:** 中国临床肿瘤学会非小细胞肺癌专家委员会

**Keywords:** 肺肿瘤, EGFR 20外显子插入突变, 规范化诊疗, 共识, Lung neoplasms, EGFR ex20ins mutations, Standardized diagnosis and treatment, Consensus

## Abstract

表皮生长因子受体（epidermal growth factor receptor, EGFR）20外显子插入（exon 20 insertion, ex20ins）突变非小细胞肺癌（non-small cell lung cancer, NSCLC）的规范化诊疗在《EGFR 20外显子插入突变非小细胞肺癌规范化诊疗中国专家共识（2023版）》中进行了阐述，提高了临床医生对该罕见靶点的关注。随着疾病领域探索的深入及新型靶向药物的获批，EGFR ex20ins NSCLC的诊疗策略也随之更新。本共识在2023版中国专家共识的基础上进行更新，专家组通过参考国内外文献及临床数据并结合专家自身临床经验，分别从疾病认知、疾病检测、疾病治疗和新型靶向药物研发现状等方面更新共识性建议，以期更好地为临床医师提供用药参考。

## Abstract (en)

The standard clinical practice of managing the non-small cell lung cancer (NSCLC) with epidermal growth factor receptor (EGFR) exon 20 insertion (ex20ins) mutations was elaborated in Chinese expert consensus on non-small cell lung cancer with EGFR exon 20 insertion mutations (2023 edition), and this rare subset has gradually attracted attention recently. With the deepening of treatment area exploration and the approval of new targeted drugs, there are more options for the diagnosis and treatment of EGFR ex20ins positive NSCLC patients. Therefore, based on the previous version of consensus, the expert panel has updated this consensus on the standardized clinical diagnosis and treatment of EGFR ex20ins mutation NSCLC through reference to literature and clinical data, and combined with the experts' own clinical experience. The updated recommendations includes disease congnition, testing methods, therapy and recent relevant clinical trials for NSCLC patients with EGFR ex20ins mutation, in order to provide better medication reference for clinical physicians.

非小细胞肺癌（non-small cell lung cancer, NSCLC）是肺癌主要的病理组织学类型，约占85%^[1]^，表皮生长因子受体（epidermal growth factor receptor, EGFR）突变是NSCLC中最常见的驱动基因，约50%的非鳞NSCLC患者携带有该基因突变^[2]^。临床实践中，NSCLC靶向治疗的疗效与基因突变亚型的关系已经得到证实^[2,3]^。EGFR 20外显子插入（exon 20 insertion, ex20ins）突变是EGFR突变的一类亚型，是继EGFR 19外显子缺失（EGFR ex19-Del）和21外显子L858R点突变（EGFR ex21-L858R）两大常见突变外EGFR的第三大突变亚型。在我国，EGFR ex20ins占所有NSCLC突变类型的0.3%-2.9%，占EGFR突变的2%-5%^[4]^。2023年发表的《EGFR 20外显子插入突变非小细胞肺癌规范化诊疗中国专家共识（2023版）》提高了临床医生对该靶点的关注度，并在如何进行规范化诊断、治疗等方面为临床实践提供了参考^[5]^。随着对更多新型化合物的深入探索以及新型靶向药物的获批，针对EGFR ex20ins NSCLC的治疗选择也在更新迭代。因此，中国临床肿瘤学会（Chinese Society of Clinical Oncology, CSCO）NSCLC专家委员会牵头，组织肺癌领域专家，参考最新国内外文献及临床数据，结合专家自身临床经验，对2023版中国专家共识进行更新，以期为各级临床医疗卫生工作者提供最新的用药参考。

**专家共识1：EGFR ex20ins是第三大EGFR突变，在我国，占EGFR突变NSCLC患者的2%-5%，异质性强，常规EGFR-酪氨酸激酶抑制剂（tyrosine kinase inhibitors, TKIs）治疗无效，患者预后差，临床需引起重视。**

在中国NSCLC患者中，EGFR突变比例约为50%^[6]^。其中常见突变包括ex19-Del和ex21-L858R，ex20ins是继ex19-Del和ex21-L858R之后的第三大突变^[7,8]^。在我国，EGFR ex20ins占所有NSCLC患者的0.3%-2.9%，占EGFR突变NSCLC患者的2%-5%^[4,9⇓⇓-12]^。根据中国开展的较大样本回顾性研究数据^[12,13]^，近年来EGFR ex20ins的发生率有所提升，占中国肺癌患者总数的2.1%-2.24%。尚未发现东西方NSCLC人群中的EGFR ex20ins发生率有显著差异。与EGFR常见突变临床特征相似，EGFR ex20ins多见于亚裔、女性、不吸烟、肺腺癌患者^[4,14]^。

EGFR ex20ins以α-C-螺旋附近密码子761与775之间框内插入和（或）复制为特征，进而导致EGFR通路激活^[15,16]^。插入位点与氨基酸序列的复杂性造就了EGFR ex20ins的强异质性。迄今为止，超过100种EGFR ex20ins亚型被报道，其中约90%发生在α-C-螺旋后的磷酸结合环，另有少部分位于α-C-螺旋的C末端（密码子761-766）。EGFR ex20ins磷酸结合环（P-Loop）内插入突变可进一步分为P-Loop近端（密码子767-772）（占ex20ins的66%-72%）与P-Loop远端（密码子773-775）（占ex20ins的27%-28%）^[17]^。在我国，已知的EGFR ex20ins亚型约85种^[13]^，中国EGFR ex20ins的真实世界研究数据^[18]^显示V769_D770ins ASV是最常见的突变亚型，占23.4%。

EGFR ex20ins的命名规则有助于临床医生准确解读基因检测报告。EGFR ex20ins可能存在不同的表达方法，无论是哪种表达形式，最终判断是否属于EGFR ex20ins的标准是20外显子的氨基酸数量是否有所增加。

对于EGFR ex20ins NSCLC患者，传统EGFR-TKIs仅对极少数亚型有效（约占EGFR ex20ins的5.1%，如A763_Y764insFQEA和A763_Y764insLQEA），而对大部分亚型治疗无效^[19]^。首先，EGFR ex20ins会诱导EGFR的α-C-螺旋和P-Loop转移至药物结合口袋，形成明显的空间位阻，导致药物结合口袋缩小，进而导致传统的EGFR-TKIs结合受阻^[20]^。其次，EGFR ex20ins构象与野生型EGFR接近，与ATP具有类似的亲和力，如何改良药物的选择性成为药物研发中的难点^[15]^。另外，插入位点结构域的不同也会影响EGFR-TKIs对EGFR ex20ins NSCLC的治疗效果，传统EGFR-TKIs对极少数位于α-C-螺旋内的突变亚型有一定疗效，而对位于P-Loop内的突变亚型疗效则十分有限^[21⇓-23]^。

截至2020年5月美国癌症电子病历数据库Flatiron Health的数据^[24]^显示，与常见EGFR突变NSCLC患者相比，EGFR ex20ins NSCLC患者的真实世界无进展生存期（real world progression-free survival, rwPFS）仅为2.9个月（vs 10.5个月，P<0.0001），真实世界总生存期（real world overall survival, rwOS）为16.2个月（vs 25.5个月，P<0.0001），EGFR ex20ins NSCLC患者预后较EGFR常见突变更差。

**专家共识2：EGFR ex20ins可采用聚合酶链式反应（polymerase chain reaction, PCR）和二代测序（next-generation sequencing, NGS）进行检测。检测样本优选组织样本；组织样本不可及时，可选择液体样本；PCR检测结果为全阴性的患者，可使用NGS进行复测。**

对于EGFR突变基因检测，技术成熟、普及性及可及性高的检测手段包括PCR和NGS，但当前传统PCR检测对于EGFR ex20ins有较高的漏检率，对EGFR ex20ins不同亚型的检测覆盖率只有19%-58%^[25]^。由于EGFR ex20ins的强异质性，传统PCR检测能力不足以覆盖所有的突变点位^[26]^。多重PCR（multiplex PCR, mPCR）在EGFR ex20ins NSCLC患者中的检测能力高于传统PCR。相关研究^[27]^显示，AmoyDx的11基因mPCR试剂盒对EGFR ex20ins阳性和阴性的诊断符合率均较高，分别为91.2%和100%。相较于PCR，NGS检测几乎可以全面覆盖EGFR ex20ins的不同亚型，敏感性及特异性更优，临床实用价值更高^[28]^。一项纳入4467例中国NSCLC患者基因检测方法的研究结果^[29]^提示NGS在EGFR ex20ins全体人群突变检出率显著高于PCR（2% vs 1.3%）。因此，基于组织样本的NGS是目前优选的检测手段，但NGS的医保覆盖率略低，费用略高，报告耗时略长^[30]^。考虑到NSCLC中国患者基数庞大，需要多样化的精准诊断手段，也需要兼顾考虑各级医院不同检测手段的可及性。对于PCR检测驱动基因全阴性的患者，建议在条件允许的情况下使用NGS进行复测^[31]^，同时，在二线治疗前应进行NGS检测。检测样本方面，首选经病理学评估合格的肿瘤组织石蜡样本或细胞学样本，但有数据^[32]^显示在约30%的晚期NSCLC患者中，采集可供检测的组织标本存在一定的困难。循环肿瘤DNA（circulating tumor DNA, ctDNA）检测有望在未来成为EGFR ex20ins诊断和疗效监测的补充手段^[33,34]^，在无法获取合格的组织或细胞学样本的情况下，可以考虑包括血浆在内的液体标本^[31]^。

**专家共识3：国内尚无一线治疗局部晚期或转移性EGFR ex20ins NSCLC的靶向药物获批，建议参考无驱动基因晚期NSCLC的一线治疗。埃万妥单抗（Amivantamab）联合化疗已获美国食品药品监督管理局（Food and Drug Administration, FDA）批准用于一线治疗局部晚期或转移性EGFR ex20ins NSCLC，国内尚未获批。对于无法耐受或拒绝化疗或体力状况（performance status, PS）评分较差等患者，一线可选择舒沃替尼（Sunvozertinib）。**

当前国内尚无针对EGFR ex20ins NSCLC一线治疗的靶向药物获批，建议参考无驱动基因的局部晚期或转移性NSCLC的一线治疗，治疗原则可参考CSCO的《非小细胞肺癌诊疗指南2024》（表1）^[35]^。

**表1:** CSCO《非小细胞肺癌诊疗指南2024》IV期无驱动基因NSCLC一线治疗

分类	分层	I级推荐	II级推荐	III级推荐
无驱动基因、非鳞NSCLC一线治疗	PS=0-1分	1. 帕博利珠单抗单药（限PD-L1 TPS≥50%，PD-L1 TPS 1%-49%）； 2. 阿替利珠单抗（限PD-L1 TC≥50%或IC≥10%）； 3. 培美曲塞+铂类联合帕博利珠单抗或卡瑞利珠单抗或信迪利单抗或替雷利珠单抗或阿替利珠单抗或舒格利单抗或特瑞普利单抗； 4. 培美曲塞联合铂类+培美曲塞单药维持治疗； 5. 贝伐珠单抗联合含铂双药化疗+贝伐珠单抗维持治疗； 6. 含顺铂或卡铂双药方案：顺铂/卡铂联合吉西他滨或多西他赛或紫杉醇或紫杉醇脂质体或长春瑞滨或培美曲塞或紫杉醇聚合物胶束。	1. 紫杉醇+卡铂+贝伐珠单抗联合阿替利珠单抗； 2. 白蛋白紫衫醇+卡铂联合阿替利珠单抗； 3. 重组人血管内皮抑制素联合长春瑞滨和顺铂+重组人血管内皮抑制素维持治疗。	纳武利尤单抗和伊匹木单抗联合2个周期培美曲塞+铂类
	PS=2分	单药化疗：吉西他滨或紫杉醇或长春瑞滨或多西他赛或培美曲塞	1. 培美曲塞+卡铂； 2. 紫杉醇周疗+卡铂； 3. 阿替利珠单抗。	
无驱动基因、鳞癌一线治疗	PS=0-1分	1. 帕博利珠单抗单药（限PD-L1 TPS≥50%，PD-L1 TPS 1%-49%）； 2. 阿替利珠单抗（限PD-L1 TC≥50%或IC≥10%）; 3. 紫杉醇+铂类联合帕博利珠单抗或卡瑞利珠单抗或舒格利单抗或派安普利单抗; 4. 紫杉醇/白蛋白紫杉醇+铂类联合替雷利珠单抗； 5. 白蛋白紫杉醇+铂类联合斯鲁利单抗； 6. 吉西他滨+铂类联合信迪利单抗; 7. 含顺铂或卡铂双药方案：顺铂/卡铂联合吉西他滨或多西他赛或紫杉醇或脂质体紫杉醇或紫杉醇聚合物胶束； 8. 含奈达铂双药方案：奈达铂+多西他赛。		1. 白蛋白紫杉醇+卡铂； 2. 纳武利尤单抗和伊匹木单抗联合2个周期紫杉醇+铂类。
	PS=2分	单药化疗：吉西他滨或紫杉醇或长春瑞滨或多西他赛	阿替利珠单抗最佳支持治疗	

CSCO：中国临床肿瘤学会；NSCLC：非小细胞肺癌；PS：体力状态；PD-L1：程序性细胞死亡配体1；TPS：肿瘤细胞阳性比例分数；TC：肿瘤细胞；IC：免疫细胞。

对于EGFR ex20ins NSCLC患者，传统治疗方案的预后相对较差，临床治疗仍存在未被满足的治疗需求。截至2020年5月，美国癌症电子病历Flatiron Health数据库的记录^[36]^显示，化疗及化疗联合方案是EGFR ex20ins NSCLC最常用的一线治疗方案（约占60%）。含铂化疗方案确认的真实世界客观缓解率（real world objective response rate, rwORR）为19.5%（n=41），中位rwPFS为5.7个月。免疫治疗联合含铂化疗确认的rwORR和中位rwPFS均劣于化疗，分别为18.8%（n=16）和4.5个月。一线治疗选择免疫单药方案的确认的rwORR和rwPFS分别为9.1%和3.1个月（n=11）；一线选择传统EGFR-TKIs治疗的rwORR和rwPFS分别为2.7%和3.3个月（n=37）。一项纳入了23个真实世界研究的系统综述及meta分析结果^[37]^显示：一线选择传统EGFR-TKIs、化疗及免疫治疗方案的ORR分别为6.8%、25.7%和14.0%；PFS分别为3.0、5.6和4.3个月。此外，研究结果提示无论是常规剂量还是双倍剂量的第三代EGFR-TKIs，都未能为EGFR ex20ins NSCLC患者带来良好获益。以上研究报道提示EGFR ex20ins NSCLC患者的一线治疗仍存在非常大的未被满足的需求。

埃万妥单抗为全人源EGFR-间质表皮转化因子（mesenchymal-epithelial transition, MET）双特异性抗体。2021年5月美国FDA基于CHRYSALIS多中心、开放性、多队列、I期研究（NCT02609776）结果加速批准埃万妥单抗单药用于治疗含铂化疗经治的EGFR ex20ins NSCLC^[38,39]^。2024年3月1日美国FDA基于PAPILLON国际多中心、随机对照、III期研究（NCT04538664）数据批准将埃万妥单抗联合化疗（卡铂+培美曲塞）用于一线治疗EGFR ex20ins NSCLC成人患者。PAPILLON研究是第一项针对EGFR ex20ins NSCLC初治人群对照标准含铂双药化疗获得阳性结果的III期研究^[40]^，宣告EGFR ex20ins NSCLC的一线治疗正式进入靶向治疗时代。该研究共纳入初治EGFR ex20ins NSCLC患者308例（埃万妥单抗联合化疗组n=153，化疗组n=155），截止2023年5月3日，中位随访14.9个月后，埃万妥单抗联合化疗组mPFS为11.4个月，显著优于化疗组的6.7个月（HR=0.4, 95%CI: 0.30-0.53, P<0.0001），显著降低患者疾病进展或死亡风险达60%，且在各亚组中保持一致，18个月PFS率分别为31%和3%。埃万妥单抗联合治疗组的ORR为73%（vs 化疗组47%，RR=1.5；95%CI：1.32-1.68；P<0.0001）。≥3级不良事件（adverse events, AEs）发生率为75%；严重AEs（serious AEs, SAEs）发生率为37%；AEs导致死亡的发生率为5%；联合组最常见的≥3级AEs包括：中性粒细胞减少症（33%）、皮疹（11%）、贫血（11%）和白细胞减少症（11%），11%的患者因AEs停用埃万妥单抗。在我国，埃万妥单抗目前正在审批中，对于中国EGFR ex20ins NSCLC患者尚不可及，2024 CSCO指南中，埃万妥单抗联合含铂双药化疗作为III级推荐。

舒沃替尼是我国自主研发的首款针对EGFR ex20ins的高选择性、不可逆的EGFR-TKI，是目前我国唯一获得EGFR ex20ins NSCLC适应证的靶向药物。舒沃替尼单药作为一线治疗药物的探索也显示出其优秀的治疗潜力。舒沃替尼一线治疗的研究数据WU-KONG 1（NCT03974022）和WU-KONG 15（NCT05559645）汇总分析结果^[41]^显示，截至2023年9月15日，舒沃替尼单药治疗的ORR达到78.6%，100%的患者观察到肿瘤缩小，其RP2D剂量组（300 mg）mPFS达到12.4个月，50%的肿瘤缓解患者仍处于持续缓解中，多个EGFR ex20ins亚型及基线伴脑转移患者中均观察到治疗反应。安全性和既往报道一致，最常见的≥3级治疗期间AEs（treatment emergent AEs, TEAEs）包括磷酸肌酸激酶（creatine phosphokinase, CPK）升高、腹泻和脂肪酶升高，大多数AEs为1-2级，临床可管理可恢复。该结果显示了舒沃替尼作为EGFR ex20ins NSCLC一线治疗的潜力。除此以外，舒沃替尼单药对比含铂化疗一线治疗EGFR ex20ins NSCLC疗效及安全性的国际多中心、III期临床研究WU-KONG 28（NCT05668988）正在进行中^[42]^。

莫博赛替尼在其对比含铂化疗一线治疗EGFR ex20ins NSCLC的III期EXCLAIM-2研究中未达到主要终点，未能显示出优于对照组（含铂化疗）的疗效^[43]^。基于此，该药物主动全球退市。

除以上靶向药物外，其他新型靶向药物一线治疗EGFR ex20ins NSCLC的相关研究疗效数据披露列于表2^[40,41,44,45]^，期待研究结果得到进一步验证。

**表2:** EGFR ex20ins NSCLC一线治疗的临床研究数据

研究	线数	分期	治疗方案	主要终点	目前状态	不良反应/安全性数据
PAPILLON^[40]^ （NCT04538664）	一线	III期	埃万妥单抗联合含铂化疗（n=153） vs 含铂化疗（n=155）	BIRC评估的PFS	进行中，截至2023年5月数据： mPFS：联合组11.4个月 vs 化疗组6.7个月 （HR=0.4, 95%CI: 0.30-0.53, P<0.0001） ORR：联合组73% vs 化疗组47% （RR=1.5, 95%CI: 1.32-1.68, P<0.0001）	≥3级AEs发生率75%；SAEs发生率37%；AEs致死发生率5%； 联合组最常见≥3级AEs：中性粒细胞减少症（33%）、皮疹（11%）、贫血（11%）和白细胞减少症（11%）
WU-KONG1（NCT03974022）、 WU-KONG15（NCT05559645） 汇总分析^[41]^	一线	I/II期	舒沃替尼 （n=28）	IRC评估的ORR	进行中，截至2023年9月数据：ORR为78.6%，mPFS（300 mg）为12.4个月	最常见≥3级TRAEs：CPK升高（17.5%）、腹泻（7%）、血脂升高（5.3%）、贫血（5.3%）、QTc间期延长（3.5%）、淀粉酶升高（3.5%）
FAVOUR（NCT04858958/CTR20201697）^[44]^	一线	Ib期	伏美替尼240 mg （n=28）	IRC评估的ORR	进行中，截至2023年6月数据： IRC评估ORR为78.6%	≥3级TRAEs发生率13%，包括口腔溃疡、QTc间期延长、白细胞减少
NCT05767866^[45]^	一线	I期	YK-029A（n=26）	安全性和耐受性	进行中，截至2022年10月31日数据：IRC评估的ORR为73.1%，mPFS为9.3个月，1年OS率为83.1%	≥3级TEAEs发生率38%， 最常见TEAEs：贫血（50.9%）、腹泻（49.1%）、皮疹（34.3%）

EGFR：表皮生长因子受体；IRC：独立评审委员会；BICR：盲态独立中心评估；mPFS：中位无进展生存期；ORR：客观缓解率；AEs：不良事件；SAEs：严重不良事件；TEAEs：治疗期间不良事件；TRAEs：治疗相关不良事件；CPK：磷酸肌酸激酶；QTc间期: 校正后QT间期；OS：总生存期。

一线治疗EGFR ex20ins NSCLC患者存在极大的未被满足的需求，EGFR ex20ins作为罕见靶点，靶向药物能给患者带来疗效的提升，且延长PFS，应作为首选。考虑埃万妥单抗国内尚无适应证且并不可及，对于无法耐受或拒绝化疗或PS评分较差的患者，一线可选择舒沃替尼。

**专家共识4：EGFR ex20ins局部晚期或转移性NSCLC患者二线治疗，优先推荐靶向治疗药物舒沃替尼。**

舒沃替尼分别于2020和2022年获得中国国家药品监督管理局（National Medical Products Administration, NMPA）、美国FDA突破性疗法认定（breakthrough therapy designation, BTD），成为肺癌领域首个获得中美两国BTD的I类新药。2023年1月舒沃替尼被NMPA纳入优先审评，同年8月正式获批，成为目前国内首个且唯一获批用于既往经含铂化疗治疗时或治疗后出现疾病进展，或不耐受含铂化疗的EGFR ex20ins阳性NSCLC成人患者的国创靶向新药，填补了临床治疗空白。推荐剂量为300 mg（2片150 mg片剂），每日一次。

舒沃替尼是基于对EGFR ex20ins的深入研究而设计的全新分子结构。在嘧啶母环C-4位采用灵活、可折叠的苯氨基结构，突破药物结合口袋空间位阻，且可以针对不同亚型进行调整灵活结合，提高对EGFR ex20ins的抑制效力和选择性，安全窗口更宽。卤素取代的苯胺基结构可占据磷酸结合环，改善药代动力学；二甲氨基吡咯烷可占据溶剂通道，进一步提高药物的物理化学性质及药代动力学^[21]^。临床前研究数据^[21]^表明，舒沃替尼对EGFR ex20ins的选择性是野生型的1.4-9.6倍，且对多种不同的EGFR ex20ins亚型、敏感突变、T790M耐药突变、罕见突变以及人表皮生长因子受体2（human epidermalgrowth factor receptor-2, HER2）20外显子插入突变均具有较好的靶向性和抗肿瘤活性。

基于舒沃替尼I/II期临床研究WU-KONG 1（NCT03974022）和WU-KONG 2（CTR20192097）^[46]^，NMPA将其纳入BTD名单。舒沃替尼的获批主要基于一项II期、单臂、多中心WU-KONG 6（CTR20211009）研究^[47]^。这项研究共纳入97例既往含铂化疗经治EGFR ex20ins NSCLC患者。截至2022年10月17日，数据分析结果基线特征部分显示，患者中位年龄58岁，32%患者伴脑转移，既往接受过一至三线的系统治疗，其中52%的患者既往接受过二线及以上治疗，35%的患者既往接受过免疫治疗，27%患者既往接受过EGFR-TKIs治疗。分子亚型方面，769_ASV突变占39%，770_SVD突变占18%，43%为其他亚型。在300 mg每日一次的推荐剂量下，独立评审委员会（Independent Review Committee, IRC）评估的ORR为61%。91%的患者肿瘤体积缩小，疾病控制率（disease control rate, DCR）为88%。对于基线伴有稳定脑转移的患者，ORR为48%。无论患者年龄、性别、吸烟史、EGFR ex20ins亚型以及既往治疗史如何，各亚组中均能观察到舒沃替尼的治疗反应。DOR、mPFS及mOS数据目前尚不成熟。安全性方面，最常见的≥3级治疗相关AEs（treatment related AEs, TRAEs）包括血清CPK升高（17%）、腹泻（8%）和贫血（6%）。舒沃替尼显示出与其他EGFR-TKIs相似的安全性。2024 CSCO指南将舒沃替尼作为经治的EGFR ex20ins NSCLC患者治疗的唯一“I级推荐”方案。

基于以上，推荐舒沃替尼为经治EGFR ex20ins NSCLC患者的优选治疗方案。EGFR ex20ins局部晚期或转移性NSCLC治疗可参考图1。

**图1:**
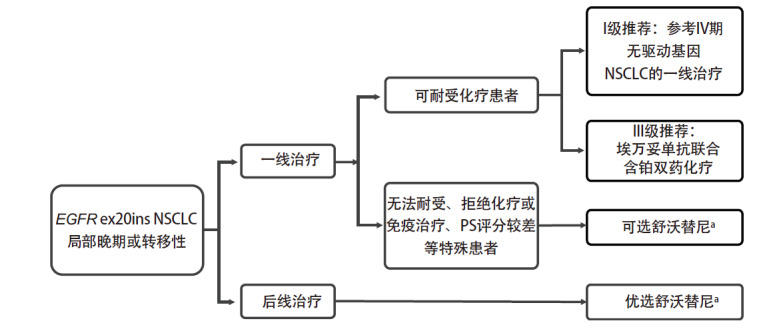
EGFR ex20ins局部晚期或转移性NSCLC治疗路径图。ex20ins：20外显子插入；^a^舒沃替尼推荐剂量为300 mg每日一次，每天同一时间服用，空腹或餐后服用均可。

**专家共识5：目前EGFR ex20ins NSCLC新型靶向药物研发主要是小分子EGFR-TKIs类药物和大分子抗体类药物。舒沃替尼AEs谱与传统EGFR-TKIs总体相似，需要关注EGFR通路常见AEs。埃万妥单抗联合化疗，除EGFR通路常见AEs外，还需注意MET通路的常见AEs和血液学毒性。推荐应重视EGFR ex20ins NSCLC患者AEs管理，早发现、早治疗。**

当前EGFR ex20ins NSCLC新型靶向药物的研发主要聚焦在小分子EGFR-TKIs类药物以及大分子抗体类药物，其毒性谱有很大的差异。舒沃替尼作为小分子EGFR-TKIs类药物的代表，其AEs与已知的EGFR-TKIs相似，多为轻症，整体可控可管理。舒沃替尼的安全性汇集（WU-KONG 1、WU-KONG 2、WU-KONG 6和WU-KONG 15）共纳入300例晚期NSCLC患者，所有患者中位暴露时间为5.6个月，接受舒沃替尼治疗的患者中，最常见的AEs（≥20%）包括皮疹、腹泻、血清CPK升高、贫血、甲沟炎、口腔黏膜炎、血肌酐升高、恶心、食欲减退、呕吐和脂肪酶升高。因TEAEs导致的治疗中断、减量或停药的患者比例分别为31.3%、20%和5.3%。在特定药物AEs中，≥3级间质性肺疾病或肺部炎症（非感染性肺炎）、QT间期延长、腹泻以及血CPK升高的发生率分别为2.7%、2.0%、9.3%和12.0%，以上事件经停药或治疗后绝大多数能好转或痊愈。

大分子抗体类靶向药物的代表是埃万妥单抗，为一款EGFR/MET靶向双特异性抗体，其常见AEs除EGFR通路相关的AEs之外，还存在MET通路相关的AEs。基于PAPILLON研究^[40]^，FDA批准埃万妥单抗联合含铂化疗应用于EGFR ex20ins NSCLC一线治疗。该研究安全性分析显示，联合治疗组最常见（≥20%）的AEs包括中性粒细胞减少、甲沟炎、皮疹、贫血、输液反应、低蛋白血症、便秘、白细胞减少、恶心、血小板减少、食欲减退、谷丙转氨酶升高、谷草转氨酶升高、痤疮样皮炎、外周性水肿、口腔黏膜炎、新冠肺炎、腹泻、低血钾、呕吐和乏力，其中≥3级AEs发生率为75%。埃万妥单抗联合化疗组因AEs导致的治疗中断、减量或停药的患者比例分别为69%、48%和24%，因AEs导致的埃万妥单抗治疗中断、减量或停药的患者比例分别为64%、36%和11%。美国FDA基于CHRYSALIS研究^[38,39]^批准其单药应用于EGFR ex20ins NSCLC二线治疗，该研究安全性数据显示其最常见（≥20%）的AEs为输液反应、甲沟炎、痤疮样皮炎、皮疹、低白蛋白血症、恶心、便秘、疲劳、口腔黏膜炎、呼吸困难、瘙痒、咳嗽、腹泻、关节痛、背部疼痛、食欲减退和周围性水肿，其中皮疹（所有级别，89%）、输液反应（所有级别，67%）是最常见的毒性反应。同时也观察到静脉血栓栓塞（venous thromboembolism, VTE）发生率为11%，间质性肺疾病的发生率为7%。因TRAEs导致的治疗中断、减量或停药的患者比例分别为29%、18%和7%^[39]^。

新型小分子靶向药物的AEs与已知的EGFR-TKIs相关AEs相似，未见新的安全信号。大分子抗体类靶向药物，需要关注其特殊AEs，如输液反应、皮肤AEs等。同时，大分子抗体类药物单药和联合化疗毒性谱有所不同，临床医生应密切关注。针对药物AEs的处理，可参考国内《EGFR-TKI不良反应管理专家共识》^[48]^和药品说明书。同时，应加强患者教育、提升早期识别和鉴别、积极管理、持续评估，进一步提升患者精准治疗的安全性管理。

**专家共识6：目前有多个针对EGFR ex20ins治疗的靶向药物正在临床研究中，可建议患者积极参与临床研究。**

目前有多个针对EGFR ex20ins NSCLC治疗的药物/新型化合物正在开展临床研究。

Zipalertinib是一种新型的口服EGFR-TKIs，以吡咯并嘧啶环为母环，可靶向EGFR ex20ins。其后线治疗I/IIa期研究（NCT04036682）截至2022年5月数据分析结果显示，73例中位治疗线数为二线的患者中，ORR为38.4%，中位随访11个月时，中位缓解持续时间（median duration of response, mDOR）为10个月，mPFS为10个月，最常见的TRAE为皮疹^[49]^。

BEBT-109是一种高活性的泛靶点EGFR抑制剂，是奥希替尼的化学结构类创新药物，目前针对EGFR ex20ins局部晚期或转移性NSCLC患者进行的多中心、开放的II期临床试验（CTR20213409）正在招募患者^[50,51]^。

当前对EGFR ex20ins NSCLC人群治疗进行探索的新型化合物还包括PLB1004^[52]^、JMT-101^[53,54]^、BLU-451^[55]^、FWD1509 MsOH^[56]^等，期待未来有更多的数据生成，增加患者的治疗选择，更好地改善EGFR ex20ins NSCLC患者的生存预后。

建议临床医生持续关注临床研究结果，可根据情况推荐患者参与（表3）。

**表3:** EGFR ex20ins NSCLC二/后线治疗的临床研究

研究	线数	分期	治疗方案	主要终点	目前状态	不良事件/安全性数据
CHRYSALIS （NCT02609776）队列4^[38,39]^	后线	I期	埃万妥单抗 （n=114）	INV评估的ORR	进行中，截至2022年9月12日数据：ORR为37%，mDOR为12.5个月，mPFS为6.9个月，mOS为23个月	≥3级AEs发生率35%；SAEs发生率30%；AEs致死发生率7%
FAVOUR （NCT04858958/ CTR20201697）^[44]^	后线	Ib期	伏美替尼240 mg （n=28） 伏美替尼160 mg （n=28）	IRC评估的ORR	进行中，截至2023年6月数据： 240 mg组ORR为46.2%；160 mg组ORR为38.5%	240 mg剂量组：≥3级TRAEs发生率29%；TRSAEs发生率8%；无TRAEs致死 160 mg剂量组：≥3级TRAEs发生率18%；TRSAEs发生率0%；无TRAEs致死
NCT04036682^[49]^	后线	I/IIa期	Zipalertinib（CLN-081） （n=73）	安全性和初步疗效	进行中，截至2022年5月9日数据：ORR为38.4%，mDOR为10个月，mPFS为10个月	≥3级TRAEs发生率23%
CTR20213409^[50,51]^	后线	II期	BEBT-109 （目标n=100）	BIRC评估的ORR	进行中	尚未披露
NCT06015503^[52]^	后线	I期	PLB1004 （n=65）	安全性和耐受性	进行中，截至2022年7月31日数据：ORR为57.7%（15/26），DCR为100%（26/26）	最常见TRAEs：腹泻（75%）、皮疹（60%）、口腔溃疡（43%）、血清肌酐升高（43%）、谷丙转氨酶升高（41%）
BECOME （NCT05132777）^[53]^	后线	II期	JMT101联合奥希替尼（n=112）	IRC评估的ORR	进行中，截至2023年6月21日数据：ORR为50%，DCR为80%	≥3级TRAEs发生率81%；TRSAEs发生率35.7%；TRAEs致死发生率3.2% 最常见TRAEs：皮疹（80%）、腹泻（68%）、食欲减退（64%）、口腔溃疡（65%）、体重降低（59%）
CONCERTO （NCT05241873）^[55]^	后线	I/II期	BLU-451 （n=19）	I期：MTD、RP2D、安全性和耐受性； II期：ORR	进行中	最常见AEs：腹泻（21%）、咳嗽（18%）、疲劳（14%）、瘙痒（14%）、皮疹（14%）， 未见≥3级AEs
NCT05068024^[56]^	后线	I期	FWD1509 MsOH （目标n=30）	安全性和耐受性	进行中	未披露

INV：研究者；mDOR：中位缓解持续时间；mOS：中位总生存期；DCR: 疾病控制率；MTD：最大耐受剂量；RP2D：推荐的II期剂量；TRSAEs：治疗相关严重不良事件。

## 结语与展望

随着EGFR ex20ins NSCLC的精准治疗受到临床上越来越多的关注以及更多新型化合物的深入探索及新型靶向药物的获批，针对EGFR ex20ins NSCLC患者的治疗选择也在更新迭代。因此，本共识专家组参考了2023版共识、最新国内外文献及临床数据，结合专家自身临床经验，形成2024版EGFR ex20ins NSCLC临床规范化诊疗专家共识。新版共识指出：首先，应当进一步重视并提高对EGFR ex20ins靶点及其疾病临床特征的认知；其次，在检测方面，推荐基于组织样本的NGS检测作为优选，组织样本不可及时可选择液体样本，对于PCR检测驱动基因全阴性的患者，建议在条件允许的情况下使用NGS进行复测；在治疗方面，当前国内尚无一线治疗EGFR ex20ins NSCLC的靶向药物获批，可参考局部晚期或转移性无驱动基因的NSCLC的一线治疗，而对于无法耐受或拒绝化疗或PS评分较差的患者，可选择舒沃替尼；EGFR ex20ins NSCLC二/后线治疗中，舒沃替尼作为国内唯一获批的口服靶向药物，填补了临床治疗上的空白，为经治的EGFR ex20ins NSCLC患者带来显著生存获益，优先推荐舒沃替尼；此外，多种新型靶向药物的临床研究正在进行中，医疗卫生工作者可持续关注相关数据并根据临床实际情况推荐患者参与临床研究。随着临床研究数据的生成及更新、新药的获批、检测技术的发展，本共识也将定期更新，以期推动EGFR ex20ins NSCLC规范化诊疗的发展，为更多中国肺癌患者带来获益。

**Table:** 

共识编写专家组成员	
**专家组组长**	
周彩存	上海市东方医院
**专家组副组长**（按姓氏汉语拼音字母排序）	
程颖	吉林省肿瘤医院
范云	浙江省肿瘤医院
王洁	中国医学科学院肿瘤医院/ 中国医学科学院肿瘤医院山西医院
王哲海	山东第一医科大学附属肿瘤医院
**执笔专家**（按姓氏汉语拼音字母排序）	
吴凤英	同济大学附属上海市肺科医院
周斐	上海市东方医院
**专家组成员**（按姓氏汉语拼音字母排序）	
褚倩	华中科技大学同济医学院附属同济医院
崔久嵬	吉林大学第一医院
董晓荣	华中科技大学同济医学院附属协和医院
郭人花	南京医科大学第一附属医院
李琳	北京医院
李伟	蚌埠医科大学
梁莉	北京大学第三医院
林根	福建省肿瘤医院
刘晓晴	307医院全军肿瘤中心
马虎	遵义医科大学第二附属医院
任胜祥	同济大学附属上海市肺科医院
沈波	江苏省肿瘤医院
王佳蕾	复旦大学附属肿瘤医院
邬麟	湖南省肿瘤医院
姚煜	西安交通大学第一附属医院
赵明芳	中国医科大学附属第一医院
周建娅	浙江大学医学院附属第一医院
